# An Account of Diseases in an Eastern District of London, from February 20, to March 20, 1809

**Published:** 1809-04

**Authors:** 


### *An Account of Diseases in an Eastern District of London, from February* 20, *to March* 20, 1809.

| | | | |
|---|---|---|---|
| ACUTE DISEASES. | | Vertigo | 2 |
| Typhus | 2 | Amenorrhœa | 3 |
| Pneumonia | 4 | Fluor Albus | 7 |
| Peripneumonia Notha | 7 | Menorrhagia | 4 |
| Pleuritis | 2 | Hypochondriasis | 2 |
| Rheumatismus Acutus | 6 | Rheumatismus Chronicus | 15 |
| CHRONIC DISEASES. | | PUERPERAL DISEASES. | |
| Dyspnœa | 9 | Menorrhagia Lochialis | 5 |
| Tussis | 14 | Dolor post Partum | 6 |
| Tussis cum Dyspnœa | 18 | Abscessus Mammæ | 1 |
| Catarrhus | 6 | Mastodynia | 5 |
| Asthma | 2 | INFANTILE DISEASES. | |
| Ascites | 2 | Convulsio | 3 |
| Hydrothorax | 4 | Erysipelas Infantile | 2 |
| Apoplexia | 1 | Diarrhœa | 4 |
| Paralysis | 3 | Pertussis | 3 |
| Cephalalgia | 4 | Herpes | 2 |

The very great change which, in a few weeks, has taken place in the temperature of the air and in the general state of the weather, has produced as remarkable a change in the state of disease. Many of those patients who were labouring under different pneumonic affections during the severe frost, and under the influence of the north-easterly winds, found themselves much relieved by the return of a more mild temperature. During a part of the present month, indeed, the cold winds have returned, and, as the consequence of it, the different complaints referred to have also recurred; but the temporary suspension of symptoms, during the favourable change of the weather, afforded a transient relief, and enabled the patients the better to struggle with a fresh though a milder attack of the disease. The season is now advancing in which attacks of this kind become less frequent, and those who have survived, and have in some measure recovered from them, may look forward to a few months reprieve. In several of the cases referred to, however, there are already some slight appearances of hydropic symptoms, and though the patient may not yet be aware of any future danger, the medical practitioner cannot fail to anticipate some alarming consequences.

